# Types of nuclear localization signals and mechanisms of protein import into the nucleus

**DOI:** 10.1186/s12964-021-00741-y

**Published:** 2021-05-22

**Authors:** Juane Lu, Tao Wu, Biao Zhang, Suke Liu, Wenjun Song, Jianjun Qiao, Haihua Ruan

**Affiliations:** 1 Tianjin Key Laboratory of Food Science and Biotechnology, College of Biotechnology and Food Science, Tianjin University of Commerce, Tianjin, China; 2 Key Laboratory of Systems Bioengineering, Ministry of Education, Tianjin, China; 3 Department of Pharmaceutical Engineering, School of Chemical Engineering and Technology, Tianjin University, Tianjin, China; 4 SynBio Research Platform, Collaborative Innovation Center of Chemical Science and Engineering, Tianjin, China

**Keywords:** Nuclear localization signal, Nuclear pore complex, Importin

## Abstract

Nuclear localization signals (NLS) are generally short peptides that act as a signal fragment that mediates the transport of proteins from the cytoplasm into the nucleus. This NLS-dependent protein recognition, a process necessary for cargo proteins to pass the nuclear envelope through the nuclear pore complex, is facilitated by members of the importin superfamily. Here, we summarized the types of NLS, focused on the recently reported related proteins containing nuclear localization signals, and briefly summarized some mechanisms that do not depend on nuclear localization signals into the nucleus.

**Video Abstract**

**Supplementary Information:**

The online version contains supplementary material available at 10.1186/s12964-021-00741-y.

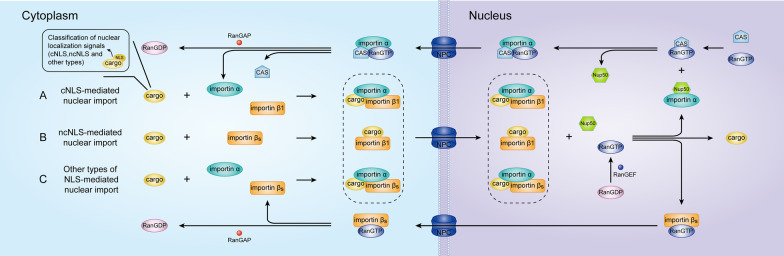

## Background

One of the characteristic features of eukaryotic cells are membrane-bound functional organelles such as the nucleus, mitochondria, golgi apparatus, and others, which are surrounded by cytoplasm. For cells to function normally, organelle proteins synthesized in the cytoplasm must be selectively and efficiently transported into their destination compartments where they can exert their physiological functions [1]. Consequently, nucleocytoplasmic transport is an essential process in eukaryotes [2, 3]. The nucleus has a double membrane called nuclear envelope. In order to allow the exchange of proteins between the nucleus and cytoplasm, proteins must be transported efficiently through the nuclear pore complex (NPC), which penetrates the nuclear envelope [4]. The NPC is a large, multimeric structure that generally acts as a permeability barrier between the cytoplasm and nucleoplasm [5]. The main structural components of the NPC include the central channel, the cytoplasmic ring moiety and cytoplasmic filaments, and the nuclear ring moiety and nuclear basket [6]. The NPC has eightfold rotational symmetry. Each NPC is connected to the inner and outer nuclear membranes by symmetrical 8 molecular spoke proteins, and the 8 molecular spoke proteins surround each other into a central channel with an outer diameter of 122 nm and an inner diameter of 70 nm [7, 8]. Diverse proteins, such as transcription factors, histones, and cell cycle regulators, need to be transported into the nucleus through the NPC after their synthesis, which necessitates the presence of a nuclear localization signal (NLS) on these cargo proteins [9]. The NLS is recognized by the corresponding nuclear transporters, which can interact with nucleoporins to help NLS-containing proteins reach the nucleus through NPCs [10]. Due to the complex roles of nuclear proteins, NLS-mediated nuclear transport is a highly regulated process. Here, we briefly review recent studies that defined NLS sequences in the nuclear import of cargo proteins and the mechanisms of protein import mediated by NLS.

### Overview of nuclear localization signals

Unlike proteins bound to the endoplasmic reticulum or mitochondria, whose N-terminal targeting signals are often cleaved after arrival at their destination organelle, nuclear localization signals remain intact and can be located at almost any part of the protein sequence, indicating the possibility of multiple rounds of nucleocytoplasmic transport [11].

A nuclear localization signal (NLS) was firstly identified through the analysis of mutants of simian virus 40 (SV40), whose NLS is composed of seven amino acids, Pro-Lys-Lys-Lys-Arg-Lys-Val (PKKKRKV) [12]. NLS sequences were subsequently identified in numerous other proteins imported into the nucleus.

In recent years, NLS were widely used in cancer treatment and viral infection prevention [13–15], and researchers paid more attention to identifying novel NLS motifs and the import nucleoporins that recognize and bind them. Here, we categorized NLS according to their residue composition into classical (cNLS), non-classical (ncNLS), and other types.

### The classical nuclear localization signals (cNLS)

As shown in Table 1, the cNLS encompass two categories, termed “monopartite” (MP) and “bipartite” (BP) [16]. MP NLS are a single cluster composed of 4–8 basic amino acids, which generally contains 4 or more positively charged residues, that is, arginine (R) or lysine (K). The characteristic motif of MP NLS is usually defined as K (K/R) X (K/R), where X can be any residue [17]. For example, the NLS of SV40 large T-antigen is ^126^PKKKRKV^132^, with five consecutive positively charged amino acids (KKKRK). Interestingly, studies have shown that its reverse sequence has no nuclear transport function [12]. Furthermore, if the third amino acid lysine (K) of SV40 large T antigen NLS is mutated to threonine (T), its nuclear transport function is also lost [18].

**Table 1:** Classification of nuclear localization signals (partial)

Category		Source	Sequence	Transport receptors
Classical nuclear localization signals (cNLS)	MP NLS	VACM-1/CUL5 [19]	**PKLKR**Q	Importin α/β1
		CXCR4 [20]	**R**P**RK**	
		VP1 [21]	**RRARRPR**G	
	BP NLS	53BP1 [22]	G**KRK**LITSEEERSPA**KRGRK**S	
		ING4 [23]	KG**KK**GRTQKEKKAA**R**A**R**S**K**GKN	
		IER5 [24]	**RKR**CAAGVGGGPAGCPAPGSTPL**KKPRR**	
		ERK5 [25]	**RKP**VTAQERQREREE**KRRRR**QERAKEREKRRQERER	
Non-classical nuclear localization signals (ncNLS)	PY-NLS	Hrp1 [26]	RSGGN**HRR**NGRGGRGGYNR**R**NNGYH**PY**	Importin β_s_
		UL79 [27]	TLLLRETMNN**LGVS**DHAVLS**R**KTPQ**PY**	
		EWS [28]	**PGKM**DKGEHRQER**R**DR**PY**	
	Other non-classical NLS	PTHrP [29]	GKKKKGKPGKRREQRKKKRRT	
		Pho4 [30]	SANKVTKNKSNSSPYLNKRKGKPGPDS	
		rpL23a [31]	VHSHKKKKIPTSPTFTTPKTLTLRRQPKYPRKSAPRRNKLDHY	
Other types of nuclear localization signals	Putative NLS	PABPN1	None	Importin α_s_/β_s_
	Spatial epitope NLS	STAT1	None	
	Cryptic NLS	FGF2	None	
	Multiple NLS	MSX1 [32]	**RK**H**K**TN**RK**P**R**	
			N**RR**A**K**A**KR**	
		NLS-RARα [33]	**R**N**KKKK**	
			**RK**VI**K**	

Willis et al*.* identified a putative NLS (^640^P**K**L**KR**Q^646^) in vasopressin-activated calcium-mobilizing protein/cullin5 (VACM-1/CUL5), which is necessary for its nuclear localization and inhibitory effect on cellular growth. This sequence starts with a proline (P) and is followed by an amino acid sequence containing three basic residues out of four (P**K**L**KR**) [19]. An analogous NLS, found in the 146th-149th amino acids of chemokine receptor CXCR4, is composed of Arg-Pro-Arg-Lys (**R**P**RK**) [20]. CXCR4 showed different subcellular distribution in pathological specimens of renal cell carcinoma derived from different sources. In primary renal cell carcinoma, it is mainly distributed in the cell membrane, while in the metastatic focus, it is mainly distributed inside the cell and nucleus [34]. Therefore, nuclear translocation of CXCR4 may be one of the important mechanisms leading to invasion and metastasis of malignant tumors such as renal cell carcinoma. Additionally, viral protein1 (VP1) of chicken anemia virus (CAV) was found to contain a functional NLS motif necessary for its import into the nucleus. It spans the amino acids ^3^**RRARRPR**G^10^, which makes it a classical MP NLS motif [21].

By contrast, BP NLS are characterized by two clusters of 2–3 positively charged amino acids that are separated by a 9–12 amino-acid linker region, which contains several proline (P) residues [16]. The consensus sequence can be expressed as R/K(X)_10-12_KRXK [17]. Notably, in BP NLS, the upstream and downstream clusters of amino acids are interdependent and indispensable, and jointly determine the localization of the protein in the cell.

For instance, the BP NLS at the C-terminus of nucleoplasmin, whose sequence is ^155^**KR**PAATKKAGQA**KKKK**^170^, can guide the protein into the nucleus. In addition to nucleoplasmin, 53BP1 (TP53-binding protein 1) also has a classical BP NLS with the sequence ^1666^G**KRK**LITSEEERSPA**KRGRK**S^1686^. Its upstream (^1667^KRK^1669^) and downstream (^1681^KRGRK^1685^) clusters are required for proper localization of 53BP1 and maintenance of genomic integrity [22, 35, 36]. ING4 contains the potential BP NLS ^128^KG**KK**GRTQKEKKAA**R**A**R**S**K**GKN^149^, among which ^142^**R**A**R**S**K**^146^ mainly binds to p53 and mediates the nuclear localization of ING4 and p53 [23]. The interaction of ING4 with p53 was abrogated in vitro and in vivo when certain mutations or deletion of the entire BP NLS domain occurred. Yamano et al*.* identified that the immediately-early response gene 5 (IER5) possess a classical BP NLS (^217^**RKR**CAAGVGGGPAGCPAPGSTPL**KKPRR**^244^), which is highly conserved among species [24], whereby both basic amino acid clusters at 217–219 and 240–244 are required for nuclear localization. The extracellular signal regulated kinase 5 (ERK5) is known to contain a classical BP NLS. Yan et al*. *[25, 37] found that the BP NLS (^505^**RKP**VTAQERQREREE**KRRRR**QERAKEREKRRQERER^539^) of ERK5 is required for the nuclear targeting of ERK5 upon activation and that this NLS itself is sufficient to drive GFP to the nucleus, indicate that the ERK5 BP NLS is biologically functional.

### Non-classical nuclear localization signals (ncNLS)

Unlike classical nuclear localization signals, many proteins have unusual NLS, which are neither similar to canonical signals nor rich in arginine or lysine residues [16]. This type of NLS is called the non-classical nuclear localization signal (ncNLS). Among them, the ncNLS of the “proline-tyrosine” category, named PY-NLS was studied in most detail.

PY-NLS is characterized by 20–30 amino acids that assume a disordered structure, consisting of N-terminal hydrophobic or basic motifs and C-terminal R/K/H(X)_2-5_PY motifs (where X_2-5_ is any sequence of 2–5 residues) [27]. Two subclasses, hPY-NLS and bPY-NLS, were defined according to their N-terminal motifs. The hPY-NLS contains φG/A/Sφφ motifs (where φ is a hydrophobic residue), whereas bPY-NLS is enriched in basic residues [27]. Collectively, the PY-NLS consensus corresponds to [basic/hydrophobic]-X_n_- [R/H/K]-(X)_2–5_-PY [38], where X can be any residue.

Human heterogeneous nuclear ribonucleoprotein A1 (hnRNP A1) is known as hPY-NLS due to its sequence ^263^FGNYNNQSSN**FGPM**KGGNFGG**R**SSG**PY**^289^, which includes a hydrophobic region (^273^FGPM^276^) required for its nuclear localization. The NLS of heterogeneous nuclear ribonucleoprotein 1 (Hrp1) closely matches the consensus of bPY-NLS (Table 1). The basic residues (^511^HRR^513^) and C-terminal R^525^ (X)_2-5_PY motif are necessary and sufficient for nuclear import, while also being required for receptor binding and protein function, respectively [26].

Wang et al. discovered the PY-NLS sequence ^66^TLLLRETMNN**LGVS**DHAVLS**R**KTPQ**PY**^92^ at the N terminus of the human cytomegalovirus protein UL79. This NLS very closely resembles a hPY-NLS, containing the C-terminal PY-core portion of the consensus sequence preceded by stretches of hydrophobic amino acids [27]. A double mutation in this PY-NLS-like sequence (P91A/Y92A) led to cytoplasmic restriction. An analogous hPY-NLS is found at the C-terminus of Ewing sarcoma (EWS) protein [28], and is required for its nuclear import. This hPY-NLS consists of 18 amino acid residues (^639^**PGKM**DKGEHRQER**R**DR**PY**^656^), among which the hydrophobic region ^639^PGKM ^642^ and C-terminal R^652^(X)_2-5_PY motif are essential. Each of the mutations R652A, P655A and Y656A could destroy the ability of the PY-NLS to direct EWS protein to the nucleus, similarly to what was observed when the whole PY-NLS was deleted [39].

However, a large number of ncNLS do not have a regular characteristic structure, including those of PTHrP (parathyroid hormone-related protein) [29], Pho4 (phosphorylation regulates association of the transcription factor) [30], and rpL23a (ribosomal protein L23a) [31], among others (Table 1).

### Other types of nuclear localization signals

In addition to the discussed cNLS and ncNLS, there also exist other types of special NLS, some of which have been studied in more detail. These include: (a) The putative NLS has basic amino acid sequence composition characteristics of nuclear localization signal. After verification, some of them have nuclear localization function, while others do not. For example, a PY-NLS-like sequence was predicted to be encoded by residues 259–306 of poly(A) binding protein nuclear 1 (PABPN1). This putative NLS is dominated by basic amino acids, but has no nuclear positioning function [38]. (b) A spatial epitope NLS was found in the primary sequence of the signal transducers and activators of transcription 1 (STAT1) [40]. This protein does not have a classical NLS, but upon dimerization, each subunit contributes basic residues that forms a cNLS, which mediates its nuclear entry [41, 42]. Such NLS cannot be recognized by the transport receptor alone, but can be recognized when several functional amino acid subunits interact. After protein–protein interaction, several basic amino acids of each subunit are close enough to each other to form a spatial structure recognized by the transport receptor, which then exerts its own nuclear positioning function [42]. (c) Cryptic NLS. Normally, proteins containing cryptic NLS cannot bind to the nuclear transport receptor, but under stimulation by specific signals, the protein structure containing the cryptic NLS can exchange to expose it, so that it can be recognized for nuclear import [43]. Min et al. identified a cryptic NLS in fibroblast growth factor 2 (FGF2). The primary amino acid sequence of low-molecular-weight (LMW) FGF2 shows that it does not have classical NLS sequence, but the apoptosis inhibitor 5 (API5) interacting region of FGF2 overlaps with its cryptic NLS region. The observations that LMW FGF2 is localized mainly in the nucleus when co-expressed with API5, but mainly in the cytoplasm when it fails to bind API5, suggested that API5 acts as a carrier protein or a stimulus signal for FGF2 trafficking into the nucleus [44]. (d) Multiple NLS. Multiple studies have shown that there is often more than one functional NLS in a single nuclear protein. For example, in the process of nuclear localization of muscle segment homeobox 1 (MSX1), Shibata et al. revealed that NLS1 (^161^**RK**H**K**TN**RK**P**R**^170^) and NLS2 (^216^N**RR**A**K**A**KR**^223^) are independently insufficient for robust nuclear localization when attached to green fluorescent protein (GFP) [32]. However, they can work cooperatively to promote nuclear localization of MSX1, leading to a significant nuclear accumulation of the corresponding GFP fusion protein. Similarly, the promyelocytic leukemia-retinoic acid receptor α (PML/RARα) has two primary NLS, which include one from the PML (^159^**R**N**KKKK**^164^), and the other from the RARα (^486^**RK**V**IK**^490^), the NLS of the RARα portion in NLS-RARα is more favorable for the nuclear localization of NLS-RARα [33, 45].

## Mechanisms of nuclear trafficking mediated by NLS

A schematic model for nuclear protein import through NPC illustrates how can a complex biological function can be generated by a spatially and temporally organized cycle of interactions between cargoes, carriers and Ran GTPase [46]. NLS-dependent protein trafficking from the cytoplasm into the nucleus is a facilitated process mediated by members of the importin (also referred to as karyopherin) superfamily [47]. Importins are further categorized into importin α_s_ and importin β_s_, based on their structural and functional features [16]. In mammalian cells, the importin α_s_ protein family only contains 6 members, namely, importin α1, importin α3, importin α4, importin α5, importin α6 and importin α7 [48, 49]. The energy for nuclear transport is provided by the small Ras family GTPases. Ran is the most abundant member of the Ras superfamily of GTPases, constituting about 0.4% of the total cell protein [50]. Ran functions as a molecular switch and undergoes a conformational change between the GDP- and GTP-bound states, with the aid of a guanine nucleotide exchange factor (RanGEF) and a GTPase-activating protein (RanGAP) [51]. Because these key regulatory factors are compartmentalized, the different forms of Ran are asymmetrically distributed in the cell, with RanGTP enriched in the nucleus and RanGDP enriched in the cytoplasm [52]. This compartmentalization allows Ran to impart directionality to nuclear transport, acting as a molecular switch that controls the compartment-specific binding and release of cargo proteins [42, 53].

### Mechanism of cNLS-mediated protein transport

Classical NLS on cargo proteins are recognized by the importin α subunit, which in turn is recognized by the importin β subunit. The resulting cNLS-cargo-importin α-importin β1 trimer is then imported into the nucleus through a series of steps [17]. This process involves the participation of multiple components as shown in Table 2 [46]. Almost all importin β_s_ contain two conserved domains, including the central HEAT domain (huntingtin, elongation factor 3 (EF3), protein phosphatase 2A (PP2A), and TOR1) and the importin β N-terminal domain (IBN) [54]. The central HEAT domain can provide binding sites for protein–protein interaction by changing its own conformation. All importin α proteins contain one importin β1 binding (IBB) domain at the N-terminal end, and the rest of the sequence contains 10 Armadillo (ARM) repeats [55, 56]. The C-terminal region of importin α is essential for the regulation of cNLS-mediated protein transport. This region acts as an interacting domain for the nuclear export factor CAS, also known as Cse1, and nucleoporin 50 (Nup50, referred to as Npap60), which catalyze cargo dissociation and function as molecular ratchets that prevent futile cycles, allowing importin α to combine with RanGTP to be exported from the nucleus [46, 57]. The binding of importin α to the nucleoporin Nup153 was reported to promote the translocation of the cNLS-cargo-importin α-importin β1 trimeric complex into the nucleus [58]. This observation is the first evidence that importin α within the trimeric complex actively contributes to the efficiency of cNLS-mediated cargo transport. These findings suggest that importin α serves not only as an adaptor molecule between the cargo and importin β1, but also actively contributes to NPC-mediated translocation by the trimeric cNLS-cargo-importin α-importin β1 complex [2, 47].

**Table 2:** Proteins related to the cNLS-mediated protein transport mechanism

Component	Other names	Function
Importin α	Karyopherin α	Adaptor that links cNLS-cargo to importin β1
Importin β	Karyopherin β	Transport factor that carries the cargo protein through the NPC
Ran	Gsp1*	Ras-superfamily GTPase that coordinates protein–protein interactions
CAS	Cse1*	A nuclear export factor dependent on importin α
RanGAP	Ran1*	GTPase-activating protein
RanGEF	RCC1	Guanine nucleotide exchange factor
Nup50	NPap60	Acts as a molecular ratchet that prevents futile cycles

^*^in *Saccharomyces cerevisiae*. *NLS* nuclear localization signal, *NPC* nuclear pore complex

The cNLS-mediated protein transport mechanism can be conveniently divided into four steps: assembly of the cargo-carrier import complex in the cytoplasm, translocation through the NPC, import-complex disassembly in the nucleus, and importin recycling [46].

Step 1: It has been demonstrated that more RanGDP protein is concentrated within the cytoplasm, and the cNLS of the cargo proteins are bound by the adaptor protein importin α, which is subsequently recognized by the carrier importin β1 to form a cNLS-cargo-importin α-importin β1 trimer. In the absence of importin β1, “NLS-like” sequences of the N-terminal IBB domain form an intramolecular bond with the NLS-binding site, inhibiting the interaction between importin α and the cNLS-cargo [59].

Step 2: Importin β1 does not directly interact with the cNLS-cargo but acts to direct importin α toward the NPC [59]. The number of NPCs in each nucleus varies depending on the organism, cell type and growth conditions. Usually, mammalian cells contain ∼3,000 to 5,000 NPCs [50]. The protein components of the NPC are known as nucleoporins (Nup). A single NPC contains 34 different Nup types, most of which are conserved among different organisms, and each Nup is represented in multiple copies [60, 61]. Interaction of importin β1 with the NPC occurs through multiple phenylalanine-glycine (FG) repeats on the Nups, enabling the trimeric complex to translocate into the nucleus [62].

Step 3: Once the trimer complex is inside the nucleus, RanGTP binding causes a conformational change in importin β1, which releases the IBB region of importin α. This autoinhibitory domain, together with Nup50 and CAS, facilitates cNLS dissociation and delivery of the cNLS-cargo in the nucleus [42].

**Fig. 1:**
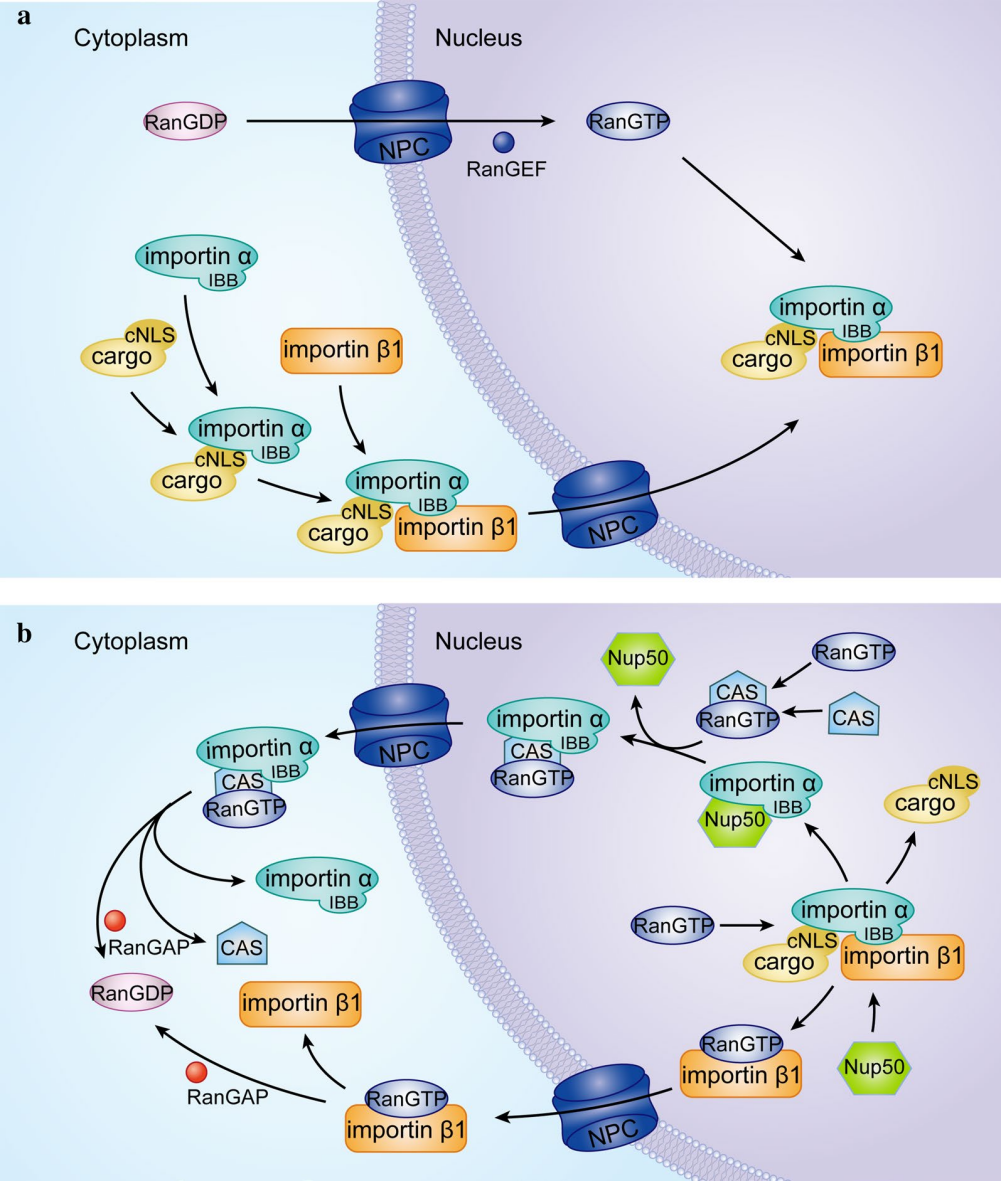
Schematic model of nucleoplasmic transport of cNLS-cargo protein. **a** Schematic model for cNLS-cargo protein import. The concentration of RanGDP protein in the cytoplasm is high, and cargo proteins with a cNLS are imported by the carrier importin β1, which binds them through the importin β1 binding (IBB) domain of the adaptor protein importin α to form the cNLS-cargo-importin α-importin β1 trimer under the action of various factors. Importin β1 directs importin α to the nuclear pore complex (NPC), and transfers the trimer complex to the nucleus by interacting with multiple phenylalanine-glycine (FG) repeats on the Nups [63]. The compartmentalization of RanGAP (GTPase activating protein) and RanEGF (guanine nucleotide exchange factor) is the basis of the proposed predominance of RanGDP in the cytoplasm and RanGTP in the nucleus [64]. **b** Schematic diagram of the cycle model of protein molecules related to nuclear transport. After passing through the NPC, the binding of RanGTP to importin β1 leads to the dissociation of importin β1 from the IBB domain of importin α. Nucleoporins such as Nup50 catalyze cargo dissociation and function as a molecular ratchet that prevents futile cycles. After dissociation, importin α is exported from the nucleus by CAS in conjunction with RanGTP. The importin β1-RanGTP complex is also returned to the cytoplasm, ready for reuse in the next round of transport

Step 4: After dissociation, importin α is exported from the nucleus by CAS in conjunction with RanGTP. The importin β1-RanGTP complex returns to the cytoplasm, where the GTP is hydrolyzed, releasing RanGDP from the importin, ready for reuse in the next round of transport [47] (Fig. 1).

### The mechanisms of ncNLS-mediated protein transport

The early studies on cNLS revolutionized the field, but it quickly became apparent that many nuclear proteins do not contain classical monopartite (MP) or bipartite (BP) NLS, and must either use alternate entry mechanisms or piggyback on cargo proteins that do contain a classical NLS [50]. One example is the abundant hnRNPA1 protein, which shuttles efficiently between the nuclear and cytoplasmic compartments, but the sequence responsible for shuttling, a PY-NLS, does not bind to importin α. Rather, hnRNPA1 was shown to be recognized by a novel protein, named importin β2, which belongs to the importin β_s_ superfamily. Vast numbers of proteins are transported into and out of the nuclear by approximately 20 species of importin β_s_ superfamily nucleocytoplasmic transport receptors [65].

In general, PY-NLS-containing cargo proteins seem to be specifically imported by importin β2 [66]. By contrast, importin β_s_-dependent cargo proteins without a PY-NLS frequently use several importin β_s_-mediated nuclear import pathways [67]. Viral, ribosomal, and histone proteins constitute the bulk of these cargo proteins [68]. The stimulated nuclear translocation of MAPKs can also directly interact with importin β_s_. It was shown that stimulated ERK directly interact with importin β7, while JNK and p38 form a trimer complex with importin β7 or importin β9 together with importin β3 and the kinase to facilitate the stimulated nuclear translocation of the latter [69]. This nuclear translocation requires the stimulated formation of heterotrimers composed of importin β3/importin β7/MAPK or importin β3/importin β9/MAPK.

It is worth noting that some proteins do not have NLS, but can use independent nuclear transport mechanisms. One example is a specific armadillo repeats of the β-catenin protein, a key mediator of Wnt signaling [70]. The β-catenin has three distinct transport sequences: the N-terminal tail, C-terminal tail and Armadillo repeats10-12 [71]. These three regions, despite sharing no apparent sequence homology, are capable of binding Nup358, Nup62, Nup98 and Nup153 of the NPC, and thus of directly mediating β-catenin entry to the nucleus via sequential and transient interactions [72]. Additionally, several publications showed that it is the direct interaction of importin β11 (IPO11) with β-catenin which mediates its nuclear import [73].

Similarly, Lyst et al. found that the nuclear protein MeCP2 (Methyl-CpG binding protein 2) may pass through the nuclear pore complex in its NLS-independent manner and import proteins, which contains two sequence-specific DNA binding motifs, AT-hook1 and methyl-CpG binding domain (MBD). Among them, an intact MBD is sufficient for nuclear localization and then be retained in the nucleus due to its affinity for DNA [74].

### Regulatory mechanisms of nuclear transport

A number of specific mechanisms precisely regulate nuclear transport in response to a variety of signals. Post-translational modification (PTM) of signaling molecules through phosphorylation/dephosphorylation is the best-understood mechanism through which nuclear transport is regulated by many different kinases/phosphatases [75, 76]. During infection by influenza A virus, the phosphorylation and dephosphorylation of Ser9 and Tyr10 controls the nuclear import of viral nucleoprotein (NP) by affecting the binding affinity between NP and different isoforms of importin α [77, 78]. Like other protein modifications, arginine methylation serves to regulate protein–protein interactions. Arginine methylation was reported to play a role in nucleocytoplasmic protein distribution by inhibiting the import of some proteins into the nucleus, or alternatively by mediating the nuclear accumulation of others [39, 79]. Mallet et al. published the first report of a functional role of arginine methylation in a cellular system by demonstrating that Arg161 methylation of poly(A)-binding protein 2 (Pab2) downregulates PY-NLS-mediated nuclear import [38]. Furthermore, acetylation was found to regulate the entry of proteins into the nucleus mainly by regulating the transport ability of importin α_s_. Bannister et al. found that the binding of Rch1 (an importin α isomer, depend on acetylase CREB binding protein (CBP)) to importin β1 was increased approximately three to fourfold after it was acetylated at Lys22 [80].

Another regulatory mechanism that controls nuclear transport relies on the binding of NLS-containing cargo proteins to specific cytoplasmic or nuclear factors that serve to anchor or retain them in cytoplasmic or nuclear compartments [75]. In the absence of ligands, the glucocorticoid receptor (GR) is retained in the cytoplasm through complexation with heat-shock protein HSP90. Upon ligand binding, GR is able to dissociate from HSP90 and is then imported into the nucleus in NLS-dependent fashion [81]. The tumor-suppressor p53 similarly appears to be retained in the cytoplasm by the ubiquitin ligase p53-associated Parkin-like cytoplasmic protein (Parc) in the absence of stress stimuli [82].

Moreover, a recent report demonstrated that arginine-rich dipeptide repeat proteins (DPRs) bind directly to importins and mediate importin condensation in a concentration- and repeat length-dependent manner [83]. This inhibits importin α/β1 and importin β_s_ mediated nuclear import. One example is that C9orf72 arginine-rich dipeptide repeat proteins (DPRs) can interact with importin β_s_, disrupt its cargo loading, and inhibit nuclear import of importin β_s_, importin α/β1, and cargoes in permeabilized mouse neurons and HeLa cells, in a manner that can be rescued by RNA [84, 85].

## Conclusions and perspectives

NLS-mediated protein import into the nucleus is an important part of nuclear and cytoplasmic information exchange in cells. At present, there have been examples of the efficiency of cross-linking peptide modification with nuclear localization signal content as vectors for intranuclear DNA delivery for the gene delivery into non-dividing cells [86]. Because of the remarkable efficiency of NLS in disease treatment, its application has become a hot topic in life sciences. Nucleocytoplasmic trafficking is functionally and mechanistically diversified, serving not only to permit operation of the basal replication, transcription, and processing machinery, but also to regulate the cell cycle, transcriptional activation and repression, circadian rhythms, and a host of other processes [50]. Classical NLS sequences have been used for artificial localization of green fluorescent protein (GFP) in the nucleus as a positioning marker, for measurement of the nuclear-cytoplasmic shuttling rate in living cells or for single molecules to track how a single protein travels through the nucleus [87–89]. In particular, many types of fluorescent proteins (FP) tagged with a NLS (FP-NLS) have been engineered as nuclear markers, as well as FP fusions with functional nuclear proteins (histone H2B, importin β et al*.*). The study of NLS can help reveal the nuclear transport mechanism of human and viral proteins [90]. Also, it can help us discover novel functions of known proteins. Studies have shown that understanding the role of the NLS in the process of parvovirus infection and its mechanism of nuclear transport can contribute to the development of therapeutic vaccines and novel antiviral medicines [91]. Although the mechanism through which importin α/β1 recognize and transport proteins with cNLS into the nucleus has been understood reasonably well, it is necessary to further explore the regulatory mechanisms of importin entry, the expression of different members of the importin family in different species and cell types, as well as the types of target proteins bound by importins. Finally, understanding the precise mechanism of translocation through the NPC remains an important future challenge.

## Data Availability

Not applicable.

## Abbreviations

NLS: Nuclear localization signal
NPC: Nuclear pore complex
SV40: Simian virus 40
cNLS: Classical NLS
ncNLS: Non classical NLS
MP: Monopartite
BP: Bipartite
VACM-1/CUL5: Vasopressin-activated calcium-mobilizing protein/cullin5
CXCR4: Chemokine receptor
VP1: Viral protein1
CAV: Chicken anemia virus
53BP1: TP53-binding protein 1
IER5: Immediately-early response gene 5
ERK5: Extracellular signal regulated kinase 5
PY-NLS: Proline-tyrosine NLS
hnRNP A1: Human heterogeneous nuclear ribonucleoprotein A1
Hrp1: Heterogeneous nuclear ribonucleoprotein 1
UL79: Human cytomegalovirus protein
EWS: Ewing sarcoma protein
PTHrP: Parathyroid hormone-related protein
Pho4: Phosphorylation regulates association of the transcription factor
rpL23a: Ribosomal protein L23a
PABPN1: Poly(A) binding protein nuclear 1
STAT1: Signal transducers and activators of transcription 1
FGF2: Fibroblast growth factor 2
LMW: Low-molecular-weight
API5: Apoptosis inhibitor 5
MSX1: Muscle segment homeobox 1
GFP: Green fluorescent protein
PML/RARα: Promyelocytic leukemia-retinoic acid receptor α
RanGEF: Guanine nucleotide exchange factor
RanGAP: GTPase-activating protein
HEAT domain: Huntingtin, elongation factor 3 (EF3), protein phosphatase 2A (PP2A), and TOR1
IBN: Importin β N-terminal domain
IBB: Importin β1 binding
ARM: Armadillo
Nup50: Nucleoporin 50
CAS: Nuclear export factor dependent on importin α
Nup: Nucleoporin
FG: Phenylalanine-glycine
PTM: Post-translational modification
MeCP2: Methyl-CpG binding protein 2
MBD: Methyl-CpG binding domain
NP: Nucleoprotein
Pab2: Poly(A)-binding protein 2
CREB: Acetylase
CBP: Acetylase binding protein
GR: Glucocorticoid receptor
Parc: Parkin-like cytoplasmic protein
DPRs: Dipeptide repeat proteins
FP: Fluorescent proteins
FP-NLS: FP tagged with a NLS
